# The Effects of Different Isocaloric Oral Nutrient Solutions on Psychophysical, Metabolic, Cognitive, and Olfactory Function in Young Male Subjects

**DOI:** 10.3389/fpsyg.2017.01988

**Published:** 2017-11-23

**Authors:** Stephan Bachlechner, Melanie Y. Denzer-Lippmann, Jan Wielopolski, Marie Fischer, Andrea Buettner, Arndt Doerfler, Christof Schöfl, Gerald Münch, Johannes Kornhuber, Norbert Thürauf

**Affiliations:** ^1^Department of Psychiatry and Psychotherapy, Friedrich-Alexander-Universität Erlangen Nürnberg, Erlangen, Germany; ^2^Department of Chemistry and Pharmacy, Emil Fischer Center, Friedrich-Alexander Universität Erlangen-Nürnberg, Erlangen, Germany; ^3^Department of Sensory Analytics, Fraunhofer Institute for Process Engineering and Packaging IVV, Freising, Germany; ^4^Department of Neuroradiology, Friedrich-Alexander-Universität Erlangen Nürnberg, Erlangen, Germany; ^5^Division of Endocrinology and Diabetes, Department of Medicine I, Friedrich-Alexander-Universität Erlangen Nürnberg, Erlangen, Germany; ^6^Department of Pharmacology, School of Medicine, University of Western Sydney, Penrith South, NSW, Australia

**Keywords:** food ingestion, food intake, oral intake, olfaction, psychophysical function

## Abstract

Food intake influences human cognition, olfaction, hunger, and food craving. However, little research has been done in this field to elucidate the effects of different nutrients. Thus, the goal of our study was to investigate the effects of oral ingestion of different nutrient solutions on olfactory, cognitive, metabolic and psychophysical function. Twenty healthy men participated in our study employing a double-blind, cross-over, repeated measurement design. Participants were tested on four different study days. Each day participants received, in randomized order, one of three isocaloric (protein, carbohydrate or fat 600 kcal, 1,500 mL) solutions or a placebo. Olfactory and cognitive tests (monitoring only) were conducted three times, i.e., 60 min before the beginning of nutrient intake, following oral ingestion of the solution and 60, and 240 min after. Psychophysical and metabolic function tests (active grehlin, desacyl ghrelin, insulin, glucagon, glucose, triglyceride, urea) were performed 7 times on each examination day (observation period: −60 min, 0 = solution intake, +60, +120, +180, +240, and +300 min). Ratings of hunger and food craving significantly differed over the observation period with lowest ratings following application of the protein solution. Highest ratings of craving were found following placebo intake. We further observed a significant positive correlation of active grehlin with hunger and fat, protein and sweets craving for each nutrient solution. Active grehlin significantly correlated with carbohydrate craving for carbohydrate and fat solution and with vegetable craving for fat solution only. Hunger hormone levels, hunger and food craving ratings demonstrated that the hierarchical order that appears in satiating efficiencies of isovolumetric-isocaloric ingested macronutrients is protein > fat > carbohydrate. Our study reveals that the type of nutrient exerts a significant influence on metabolic parameters, hunger and food craving.

## Introduction

Food intake is controlled by short-acting and long-acting regulatory mechanisms. The aim of the long-acting regulation is to control fat resources to maintain body weight (Wilding, 2002). The short-acting regulation is important for daily food intake. When food is ingested, the stomach and the small intestine expand, which can be measured by mechanosensors (Gekle et al., 2010). These sensors send information about the gastrointestinal expansion to the nucleus tractus solitarii, which inhibits the hunger center (Berthoud and Neuhuber, 2000).

A pure mechanical gastric distension study performed by Wang et al. (2008) provided evidence that the left amygdala and insula process interoceptive signals of fullness produced by gastric distention involved in the control of food intake. Additionally, researchers showed that water intake a short time before eating reduces hunger and leads to weight reduction (Parretti et al., 2015). However, Oesch et al. (2006) observed that transient pure mechanical distension of the fundus or the antrum prior to a meal does not trigger satiation. Moreover, Mion et al. (2005) observed in obese subjects that gastric emptying rates and plasma ghrelin levels were decreased in the presence of an intragastric balloon. The researchers also reported that the presence of the balloon in the stomach was associated with a significant decrease in ghrelin secretion, despite the concomitant weight loss. Another isovolumetric gastric distension study by Wijlens et al. (2016) showed that a high caloric gastric infusion increased satiety and reduced subsequent energy intake compared to an isovolumetric low caloric gastric infusion. However, different macronutrients were not tested separately.

Several investigations indicate the effect of isovolumetric and isocaloric food intake on hunger and satiety (Cecil et al., 1999; St-Onge et al., 2004; Wijlens et al., 2012). Researchers showed the importance of orosensory stimulation in combination with gastric stimulation of food ingestion on satiety (Cecil et al., 1999; Wijlens et al., 2012). In an oral and gastric manipulation study with i.a. an isovolumetric and isocaloric food intake, researchers found that only the combination of oral and gastrointestinal food application leads to decreased energy intake (Wijlens et al., 2012). Cecil et al. (1999) demonstrated that oral administration of a high fat meal induces a greater effect on appetite and slows gastric emptying more than an isovolumetric and isocaloric high-level carbohydrate meal. Thus, high fat meal would have the effect of prolonging gastric distension. Further, St-Onge et al. showed that an isovolumetric and isocaloric mixed nutrient beverage leads to higher satiation over time compared to a sugar-only beverage (St-Onge et al., 2004). The type of macronutrient and the energy per time ratio also represent important factors affecting satiety as well as psychophysical, metabolic, cognitive and olfactory function. Many studies have demonstrated that fat ingestion has a relatively weak impact on satiety; as a consequence, a high fat diet leads to weight gain because more food has to be consumed to feel satiated (de Castro, 1987; Lissner et al., 1987; Bellisle et al., 1998). Several researchers observed that protein intake suppresses subsequent food consumption and has a higher satiating effect than fat and carbohydrate (de Castro, 1987; Poppitt et al., 1998).

Further, Macht et al. (2003) demonstrated that, with increasing energy density of food, negative emotions like anger, fear, sadness and shame are induced directly after consumption. Additionally, it was shown that carbohydrate ingestion leads to lower depression scores compared to protein ingestion in healthy subjects (de Castro, 1987; Fischer et al., 2001).

Besides, human olfaction is also influenced by food and eating. Many researchers observed changes in olfactory detection thresholds depending on food intake (Guild, 1956; Furchtgott and Friedman, 1960; Berg et al., 1963; Albrecht et al., 2009; Stafford and Welbeck, 2011; Ramaekers et al., 2016). For example, Ramaekers et al. (2016) showed that hunger enhanced sensitivity to food odors, but the researchers did not observe sensory-specific satiety.

In addition, several studies have shown a relation between the ingestion of glucose and improved memory performance: Administration of glucose can facilitate verbal declarative memory in healthy young adults and teenagers (Foster et al., 1998; Suenram-Lea et al., 2004; Smith et al., 2009), and also in older persons (Kaplan et al., 2001; Riby et al., 2004). Jones et al. (2012) found that different macronutrients elicit different patterns of this effect over time. The researchers suggested that the impact of different macronutrients on cognition may be related to nutrient-specific mechanisms.

The different findings of nutrient effects on psychophysical, metabolic and olfactory functions reported so far motivated our study investigating these effects for isocaloric-isovolumetric oral nutrient solutions. We hypothesize that the ingestion of different isocaloric macronutrients will dissimilarly affect psychophysical, metabolic and olfactory functions because of different metabolic pathways. Further, we hypothesize that ingestion of a placebo solution will also affect psychophysical parameters due to distension of the stomach. In order to test our hypotheses we conducted a study investigating the influence of different orally applied macronutrients (protein, carbohydrate and fat) and placebo on psychophysical, metabolic, olfactory and cognitive (monitoring only) functions.

## Methods

### Participants

Twenty healthy young male volunteers with a mean BMI of 22.79 ± 1.44 kg/m^2^ participated in this study (age range: 20–32 years, mean age: 23.80 ± 3.22 years).

Exclusion criteria consisted of severe psychiatric illness, judged by SCID-interview (structured clinical interview for DSM-IV) and BDI (Beck-Depressions-Inventory), vegan lifestyle or unusual eating habits (FEV-questionnaire regarding eating behavior; this questionnaire is used to check for normal eating behavior. It asks for symptoms of binge eating and other eating disorders). We used this questionnaire to ensure that our subjects have no eating disorders. Further exclusion criteria were somatic illness and abnormal hemogram, drug use, known intolerance or allergic reaction to substances contained in the nutrient solutions, smoking, BMI > 25 kg/m^2^, and age under 18 and over 45 years. Females were excluded from our study due to hormonal changes during menstrual cycles that are likely associated with changes in psychophysical parameters (Cohen et al., 1987; Weingarten and Elston, 1991; Hill and Heaton-Brown, 1994).

All volunteers fulfilling none of these criteria were included. Volunteers were recruited via the homepage of the university clinical center and via bulletins on community boards at the Friedrich-Alexander-Universität Erlangen-Nürnberg. All experimental procedures were clearly explained, and volunteers provided written informed consent prior to the testing sessions. Participants were free to interrupt and terminate the testing sessions at any time. This study was carried out in accordance with the recommendations of the Declaration of Helsinki with written informed consent from all subjects. All subjects gave written informed consent in accordance with the Declaration of Helsinki. The protocol was approved by the Ethics Committee of the Friedrich-Alexander-Universität Erlangen-Nürnberg.

### Design

**Figure 1:**
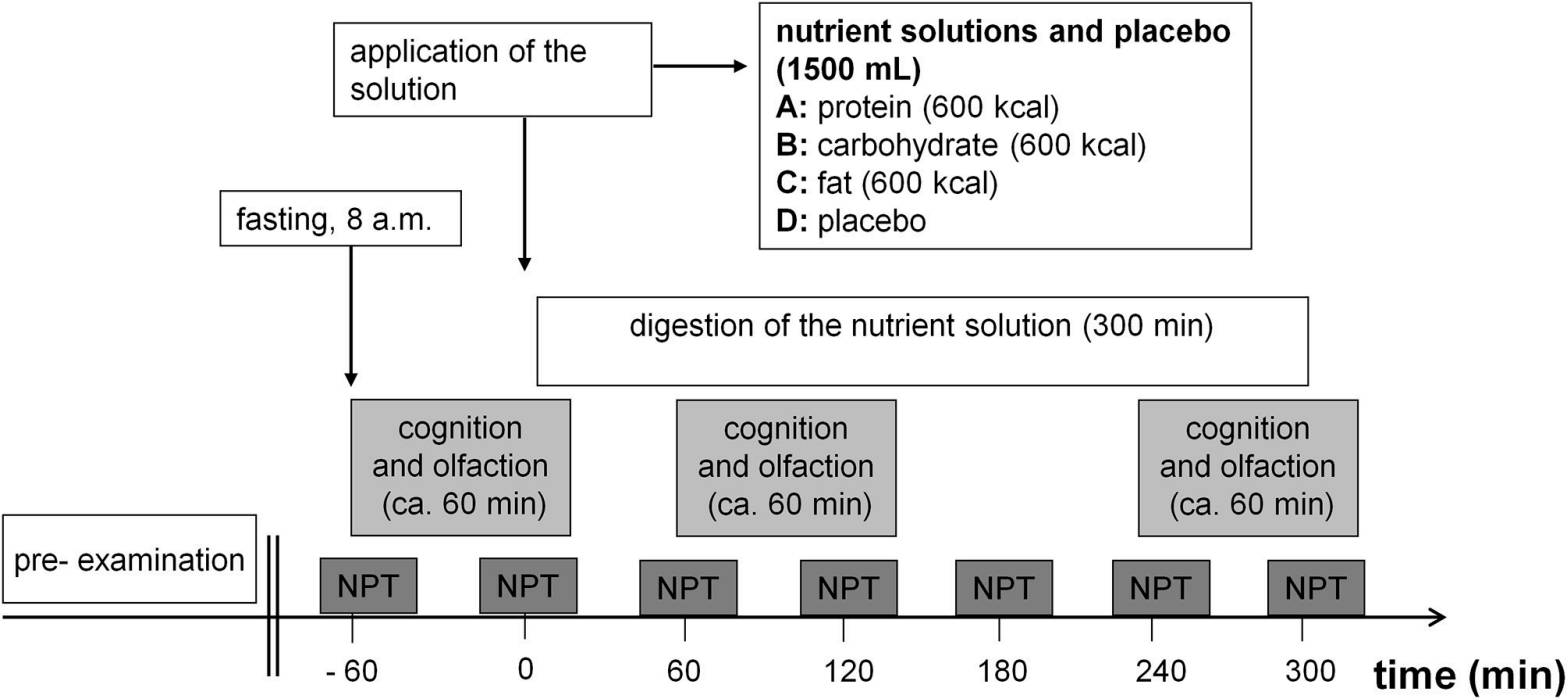
Study design. 08:00 start of the examination day. Dark gray boxes, Registering of alertness, psychophysical parameters (mood, hunger, fat craving, protein craving, carbohydrate craving, sweets craving, and vegetables craving) and collecting of blood samples for measuring metabolic parameters (active ghrelin, desacyl ghrelin, glucose, insulin, glucagon, triglycerides, urea). Test sessions that included a cognitive test (alertness, working memory, incompatibility) and an olfactory test (*n*-butanol threshold, discrimination, and identification) were performed in pre-intake status and in post-intake status. Participants ingested a different solution depending on examination day (A) protein solution, (B) carbohydrate solution, (C) fat solution, or (D) placebo solution.

A randomized, double-blind, cross-over, repeated measurement design was employed for the study. The study consisted of four testing days with a 4–10 inter-day period. On the different testing days, participants consumed different nutrient solutions (protein, carbohydrates, fat) or placebo within 30 min. The application order was randomized, i.e., each 25% of the panelists started with a protein, carbohydrate, fat or placebo solution. Figure 1 shows the study design including all test sessions and all parameters tested. Note that cognitive and olfactory testing were executed three times starting 60 min before intake of the nutrient solution (pre-intake status following fasting overnight) (time 1), 60 min (time 2) and 240 min (time 3) after the beginning of the intake of the nutrient solutions (post-intake status). First, subjects completed the cognitive testing (testing time: about 20 min), followed by the olfactory testing (testing time: about 40 min).

### Solutions

Four isovolumetric (1,500 mL) and isoenergetic (600 kcal) nutrient solutions and placebo (1,500 mL) were administered. The drinks were administered in opaque cups, covered by lids. The solutions were freshly-prepared in the kitchen and stored in the refrigerator until consumption. The formulations of the different solutions are listed in Table 1. Intake of the solutions was established by drinking a volume of 1,500 mL within 30 min (1,500 mL/30 min).

**Table 1:** Formulation of the different nutrient solutions and placebo solution.

**Ingredients**	**Protein**	**Carbohydrate**	**Fat**	**Placebo**
Inulin (Spinnrad GmbH, Segeberg)	1.5 g	1.5 g	1.5 g	1.5 g
Carboxymethyl cellulose (Dagmar Köhler, Alpen)	3.0 g	3.75 g	3.0 g	3.75 g
Glucose (Sigma Aldrich Chemie GmbH, Steinheim)	–	63.98 g	–	–
Maltodextrin (Sigma Aldrich Chemie GmbH, Steinheim)	–	92.74 g	–	–
Whey protein (Iron Maxx Sporternährung, Köln)	157.83 g	–	–	–
Liquigen (Nutricia GmbH, Erlangen)	–	–	109.59 mL	–
Lecithin (Spinnrad GmbH, Segeberg)	–	–	15 g	–
Aspartame (Acros Organics, Belgium)	0.225 g	–	0.225 g	0.225 g
Food dye white (pati-Versand GmbH, Herzlake)	10 mL	10 mL	10 mL	10 mL
Caramel flavor (Dagmar Köhler, Alpen)	1.5 mL	3 mL	3 mL	3 mL
Water (Evian, Danone Waters Deutschland GmbH)	To achieve a volume of 1,500 mL	To achieve a volume of 1,500 mL	To achieve a volume of 1,500 mL	To achieve a volume of 1,500 mL

### Psychophysical function

All psychophysical functions were tested shortly before blood samples were collected from the participants. Subjects rated “hunger,” and “food craving” employing VAS (visual analog scales, ranging from −10 to +10, including 0 as a neutral point). Food cravings were rated following the presentation of five pictures (order of pictures: 1. fat-rich food, 2. protein-rich food, 3. carbohydrate- rich food, 4. sweets, 5. vegetables). Each visual presentation lasted 5 s. Mood was rated using the Kunin scale (Kunin, 1955). This is an ordinal scale and measures the non-numeric concept of happiness employing seven different faces expressing the status “very happy,” “happy,” “little happy,” “neutral,” “little sad,” “sad,” “very sad.” Subjects had to choose one of the seven faces to describe their current mood.

### Metabolic function

At the beginning of each testing day an i.v. line was initiated for blood sample collection for each participant. To obtain blood plasma, blood samples were collected in tubes (Sarstedt AG & CoKG, Nümbrecht, Germany) that contained NaF (1.0 mg/mL blood) and EDTA (1.2 mg/mL blood). To obtain blood serum, blood samples were collected in tubes (Sarstedt AG & CoKG, Nümbrecht, Germany) that contained coagulation activators. HCl or protease inhibitors were not added to the blood samples. Thus, it is possible that the reported ghrelin concentrations are lower than those that would actually be circulating in the subjects.

Active Ghrelin: Blood plasma active ghrelin was determined by a two-site sandwich enzyme-linked immunosorbent assay (ELISA) (Biotrend Chemikalien GmbH, Germany, Cologne) (inter-assay CV: 0.069).

Desacyl ghrelin: Blood plasma desacyl ghrelin was determined by a two-site sandwich enzyme-linked immunosorbent assay (ELISA) (Biotrend Chemikalien GmbH, Germany, Cologne) (inter-assay CV: 0.077).

Insulin: The blood serum insulin was determined by chemiluminescent immunoassay technology using LIAISON Insulin (DiaSorin Deutschland GmbH, Germany, Dietzenbach).

Glucagon: Blood plasma glucagon was determined by a competitive enzyme immunoassay (EIA) (Biotrend Chemikalien GmbH, Germany, Cologne) (inter-assay CV: 0.073–0.189).

Glucose: Blood plasma glucose was determined by photometric measurement techniques via hexokinase method using AU5800 Clinical Chemistry System (Beckman Coulter GmbH, Germany, Krefeld).

Triglyceride: Blood serum triglyceride level was determined by photometric measurement techniques via the colorimetric method using AU5800 Clinical Chemistry System (Beckman Coulter GmbH, Germany, Krefeld).

Urea: Blood serum urea level was determined by photometric measurement techniques via the kinetic measurement of urease using AU5800 Clinical Chemistry System (Beckman Coulter GmbH, Germany, Krefeld).

### Cognitive function

All cognitive tests were performed on a computer using the Tests for Attentional Performance 2.2 (Vera Fimm, Herzogenrath, Germany).

Ratings of alertness: Subjects rated “alertness” employing visual analog scales (ranging from −10 maximal tiredness, to 10, maximal alertness, including 0 as a neutral point).

Alertness (with and without warning tone), working memory (advanced version) and incompatibility were tested according to the instruction manual (Verafimm, 2016).

### Olfactory function

For olfactory testing (*n*-butanol threshold, discrimination, identification), the well-validated (Kobal et al., 2000; Denzer et al., 2014) Sniffin' Sticks test battery (Hummel et al., 1997) (Burghart Messtechnik GmbH, Wedel, Germany) was used in a counterbalanced order. During the test the examiners wore odorless gloves.

For the threshold test we used a single up-down staircase method (Hummel et al., 1997). The pens of the identification test were additionally evaluated:

After the pen was identified, subjects were asked to rate the intensity (20 cm scale, 0 very low intensity, 20 very high intensity) and the pleasantness (−10 to 10 cm scale, −10 very unpleasant, 10 very pleasant) of the odor on an analog rating scale. Hedonic ratings were registered at the end of time 1, 2 and 3 (pre-ingestion and about 120 min, about 300 min after application of the nutrient solution, respectively), i.e., at the end of the olfactory testing session (time 2, 3) as well as directly before ingestion of the nutrient solution, i.e., at the end of the first olfactory testing session (time 1).

### Statistical analyses

Data were analyzed using SPSS (version 22.0 for Windows, SPSS IBM). We tested for normal distribution employing the Shapiro Wilk test. Mauchly's test was used to measure sphericity. If sphericity was violated, Greenhouse–Geisser corrections were applied. To compare olfaction and cognition in pre-intake and 60 min and 300 min post-intake status depending on the solutions, and to compare each of the seven measurement points of alertness and the psychophysical and metabolic parameters depending on the solutions, our data were subjected to a two-way repeated-measurement analysis of variance (ANOVA) with “time” and “solution” as within-subjects factors. The Bonferroni test was used for *post-hoc* testing. In the case of non-normal distribution, non-parametric testing was executed employing the Friedman test and the Wilcoxon *post-hoc* test. To test the effect of placebo alone we used a one-way repeated-measurement ANCOVA with “time” as within-subjects factor. The Bonferroni test was used for *post-hoc* testing (psychophysical and metabolic parameters: base = sessions 2, 0 min vs. post-intake = session 3–7, 60, 120, 180, 240, and 300 min; cognitive and olfactory parameters: base = session 1, −60 min vs. post-intake = session 2, 3, 60, and 240 min). For comparison of the different solutions at each measurement point (1–7 for psychophysical and metabolic factors and 1–3 for cognitive and olfactory factors), we used a one-way repeated-measurement ANCOVA with “solution” as within-subjects factor. The Bonferroni test was used for *post-hoc* testing.

Base-to-Peak analyses of psychophysical and metabolic parameters: To compare each measurement point of the psychophysical and metabolic parameters (post-intake to pre-intake; base = sessions 2, 0 min for psychophysical and metabolic parameters), we employed paired *t*-tests for all conditions separately (protein, carbohydrate, fat, and placebo).

Pearson correlation coefficients were calculated to estimate the correlation between ghrelin concentrations (active ghrelin and desacyl ghrelin) and their hunger and food craving (fat, protein, carbohydrate, sweets, and vegetables) measures after exposure to each solution (protein, carbohydrate, fat and placebo).

## Results

### Psychophysical function

#### Mood

Placebo: The factor “time” had no significant effect on “mood” [*F*_(1, 6)_ = 0.73, *p* = 0.55].

**Figure 2:**
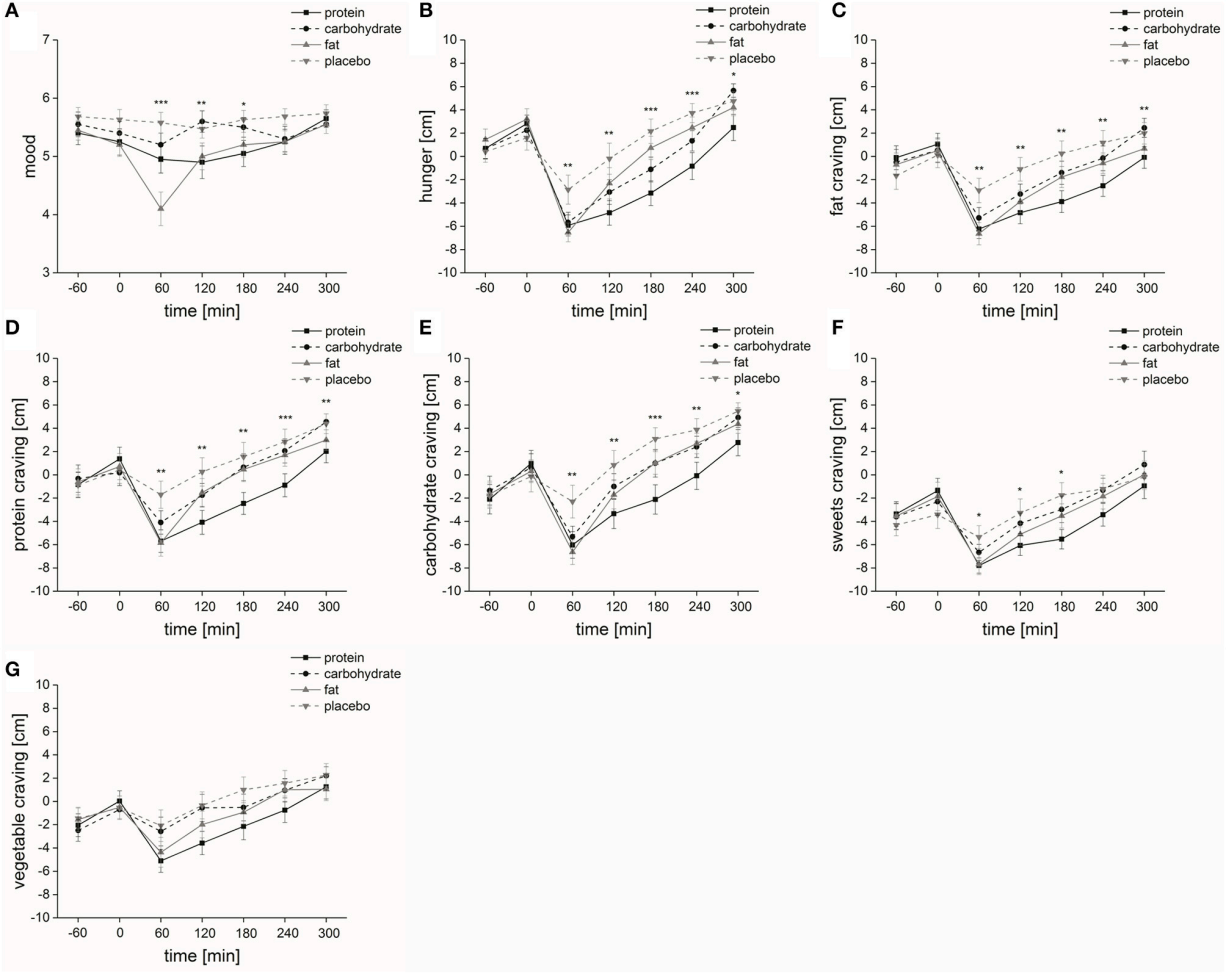
Psychophysical parameters. Time course and standard errors of means of the mean psychophysical parameters **(A)** mood, **(B)** hunger, **(C)** fat craving, **(D)** protein craving, **(E)** carbohydrate craving, **(F)** sweets craving, and **(G)** vegetable craving of all participants (*n* = 20) during application of the four different solutions (protein, carbohydrate, fat, or placebo). Statistical significance: ^*^p ≤ 0.05; ^**^p ≤ 0.01; ^***^p ≤ 0.001.

Macronutrient and placebo solution: The factors “time”, “solution” and “time × solution” had a significant impact on “mood” [“time”: *F*_(1, 6)_ = 6.4, *p* ≤ 0.001; “solution”: *F*_(1, 3)_ = 9.9, *p* ≤ 0.001; “time × solution”: *F*_(3, 6)_ = 3.9, *p* ≤ 0.001]. The comparison of mood at each measurement point showed that at time 3 [*F*_(1, 3)_ = 20.6, *p* ≤ 0.001], time 4 [*F*_(1, 3)_ = 6.8, *p* ≤ 0.01] and time 5 [*F*_(1, 3)_ = 4.9, *p* ≤ 0.05] mood significantly differed between the four solutions (Figure 2A).

Isovolumetric conditions: *Post-hoc* analyses demonstrated that mood ratings were significantly higher regarding placebo intake compared to protein (*p* ≤ 0.05) and fat (*p* ≤ 0.001) intake. At time 3, *post-hoc* analyses demonstrated that mood ratings were significantly higher after placebo intake compared to protein (*p* ≤ 0.001) and fat (*p* ≤ 0.001) intake. *Post-hoc* analyses at time 5 did not show any differences between the four solutions.

Isovolumetric-isocaloric conditions: *Post-hoc* analyses demonstrated that mood ratings were significantly lower regarding fat intake compared to carbohydrate intake (*p* ≤ 0.05). At time 3, *post-hoc* analyses demonstrated that mood ratings were significantly lower after fat intake compared to protein (*p* ≤ 0.001) and carbohydrate (*p* ≤ 0.01) intake. At time 4, *post-hoc* analyses demonstrated that mood ratings were significantly higher after carbohydrate intake compared to protein (*p* ≤ 0.05) and fat (*p* ≤ 0.01) intake.

#### Hunger

Placebo: The factor “time” significant affected “hunger” [*F*_(1, 6)_ = 14.4, *p* ≤ 0.001]. *Post-hoc* analyses demonstrated that hunger ratings were significantly higher at time 2 compared to time 3 (*p* ≤ 0.05). Differences in base-to-peak ratios (time 2 compared to time 3–7) for placebo are presented in Table 2.

**Table 2:** Placebo solution: Numerical difference of means of the psychophysical parameters.

	**Mood**		**Hunger**		**Fat craving**		**Protein craving**		**Carbohydrate craving**		**Sweets craving**		**Vegetable craving**	
	**Difference**	***P*-value**	**Difference (cm)**	***P*-value**	**Difference (cm)**	***P*-value**	**Difference (cm)**	***P*-value**	**Difference (cm)**	***P*-value**	**Difference (cm)**	***P-*value**	**Difference (cm)**	***P*-value**
m3-m2	−0.053	0.72	−4.5	**0.002**	−3.0	**<0.000**	−2.1	0.053	−2.2	**0.041**	−1.9	**0.036**	−1.5	0.22
m4-m2	−0.16	0.33	−1.8	0.18	−1.2	0.16	−0.18	0.86	0.97	0.38	0.14	0.87	0.21	0.84
m5-m2	0	1.0	0.56	0.57	0.13	0.83	1.1	0.33	3.2	**0.006**	1.7	0.085	1.6	**0.043**
m6-m2	0.053	0.67	2.1	**0.026**	1.1	0.13	2.4	**0.033**	4.0	**0.003**	2.2	0.052	2.1	**0.010**
m7-m2	0.11	0.49	3.1	**0.004**	1.9	**0.006**	4.0	**0.001**	5.6	**<0.000**	3.2	**0.004**	2.8	**0.001**

*m, mean of session; significant differences are presented in bold*.

Macronutrient and placebo solution: The factors “time,” “solution,” and “time × solution” had a significant impact on “hunger” [“time”: *F*_(1, 6)_ = 40.7, *p* ≤ 0.001; “solution”: *F*_(1, 3)_ = 8.6, *p* ≤ 0.001; “time × solution”: *F*_(3, 6)_ = 4.7, *p* ≤ 0.001]. *Post-hoc* analyses demonstrated that hunger ratings were significantly lower regarding protein intake compared to fat (*p* ≤ 0.05) and placebo (*p* ≤ 0.001) intake and significantly lower regarding carbohydrate intake compared to placebo intake (*p* ≤ 0.05). The comparison of hunger at each measurement point showed that at time 3 [*F*_(1, 3)_ = 5.4, *p* ≤ 0.01], time 4 [*F*_(1, 3)_ = 6.7, *p* ≤ 0.01], time 5 [*F*_(1, 3)_ = 9.6, *p* ≤ 0.001], time 6 [*F*_(1, 3)_ = 8.7, *p* ≤ 0.001], and time 7 [*F*_(1, 3)_ = 5.3, *p* ≤ 0.05] hunger significantly differed between the four solutions (Figure 2B).

Isovolumetric conditions: At time 3, *post-hoc* analyses demonstrated that hunger ratings were significantly higher after placebo intake compared to fat intake (*p* ≤ 0.05). At time 4, *post-hoc* analyses demonstrated that hunger ratings were significantly higher after placebo intake compared to protein (*p* ≤ 0.001) and carbohydrate (*p* ≤ 0.05) intake. At time 5, *post-hoc* analyses demonstrated that hunger ratings were significantly higher after placebo intake compared to protein (*p* ≤ 0.001) and carbohydrate (*p* ≤ 0.001) intake. At time 6, *post-hoc* analyses demonstrated that hunger ratings were significantly higher after placebo intake compared to protein (*p* ≤ 0.001) and carbohydrate (*p* ≤ 0.05). *Post-hoc* analyses at time 7 did not show any differences between the four solutions.

Isovolumetric-isocaloric conditions: At time 5, *post-hoc* analyses demonstrated that hunger ratings were significantly lower after protein intake compared to fat intake (*p* ≤ 0.05). At time 6, *post-hoc* analyses demonstrated that hunger ratings were significantly lower after protein intake compared to fat intake (*p* ≤ 0.05).

#### Food craving

Placebo: Food craving was significantly affected by the factor “time” [fat-rich food: *F*_(1, 6)_ = 10.4, *p* < 0.001; protein-rich food: *F*_(1, 6)_ = 10.8, *p* < 0.001; carbohydrate-rich food: *F*_(1, 6)_ = 18.1, *p* < 0.001; sweets: *F*_(1, 6)_ = 9.3, *p* < 0.001; vegetables: *F*_(1, 6)_ = 7.1, *p* < 0.001]. *Post-hoc* analyses demonstrated that fat craving was significantly higher at time 2 compared to time 3 (*p* ≤ 0.01). *Post-hoc* analyses demonstrated that protein craving was significantly lower at time 2 compared to time 7 (*p* ≤ 0.05). *Post-hoc* analyses demonstrated that carbohydrate craving was significantly lower at time 2 compared to time 7 (*p* ≤ 0.01). *Post-hoc* analyses demonstrated that vegetable craving was significantly lower at time 2 compared to time 7 (*p* ≤ 0.05). Differences in base-to-peak ratios (time 2 compared to time 3–7) for placebo are presented in Table 2.

Macronutrient and placebo solution: Food craving was significantly affected by the factor “time” [fat-rich food: *F*_(1, 6)_ = 22.5, *p* < 0.001; protein-rich food: *F*_(1, 6)_ = 23.8, *p* < 0.001; carbohydrate-rich food: *F*_(1, 6)_ = 27.5, *p* < 0.001; sweets: *F*_(1, 6)_ = 18.6, *p* < 0.001; vegetables: *F*_(1, 6)_ = 10.8, *p* < 0.001]. With the exception of sweets [*F*_(1, 3)_ = 2.3, *p* = 0.11], food craving was significantly affected by the factor “solution” [fat-rich food: *F*_(1, 3)_ = 6.2, *p* < 0.01; protein-rich food: *F*_(1, 3)_ = 8.6, *p* < 0.001; carbohydrate-rich food: *F*_(1, 3)_ = 9.4, *p* < 0.001; vegetables: *F*_(1, 3)_ = 5.2, *p* < 0.01]. With the exception of vegetable craving [*F*_(3, 6)_ = 2.0, *p* = 0.079], “time × solution” interaction also significantly influenced food craving [fat-rich food: *F*_(3, 6)_ = 4.2, *p* < 0.001; protein-rich food: *F*_(3, 6)_ = 3.1, *p* < 0.01; carbohydrate-rich food: *F*_(3, 6)_ = 3.5, *p* < 0.001; sweets: *F*_(3, 6)_ = 2.8, *p* < 0.01] (Figures 2C–G). The comparison of fat craving at each measurement point showed that at time 3 [*F*_(1, 3)_ = 6.2, *p* ≤ 0.01], time 4 [*F*_(1, 3)_ = 6.0, *p* ≤ 0.01], time 5 [*F*_(1, 3)_ = 6.9, *p* ≤ 0.001], time 6 [*F*_(1, 3)_ = 7.3, *p* ≤ 0.001] and time 7 [*F*_(1, 3)_ = 6.0, *p* ≤ 0.01] there were significant differences between the four solutions (Figure 2C). The comparison of protein craving at each measurement point showed that at time 3 [*F*_(1, 3)_ = 7.5, *p* ≤ 0.01], time 4 [*F*_(1, 3)_ = 6.3, *p* ≤ 0.01], time 5 [*F*_(1, 3)_ = 5.4, *p* ≤ 0.01], time 6 [*F*_(1, 3)_ = 8.0, *p* ≤ 0.001], and time 7 [*F*_(1, 3)_ = 6.0, *p* ≤ 0.01] there were significant differences between the four solutions (Figure 2D). The comparison of carbohydrate craving at each measurement point showed that at time 3 [*F*_(1, 3)_ = 7.2, *p* ≤ 0.001], time 4 [*F*_(1, 3)_ = 7.1, *p* ≤ 0.001], time 5 [*F*_(1, 3)_ = 9.8, *p* ≤ 0.001], time 6 [*F*_(1, 3)_ = 6.8, *p* ≤ 0.01], and time 7 [*F*_(1, 3)_ = 4.4, *p* ≤ 0.05] there were significant differences between the four solutions (Figure 2E). The comparison of sweets craving at each measurement point showed that at time 3 [*F*_(1, 3)_ = 4.4, *p* ≤ 0.05], time 4 [*F*_(1, 3)_ = 3.1, *p* ≤ 0.05], and time 5 [*F*_(1, 3)_ = 4.2, *p* ≤ 0.05] there were significant differences between the four solutions (Figure 2F).

Isovolumetric conditions: *Post-hoc* analyses demonstrated that fat craving was significantly higher regarding placebo intake compared to protein intake (*p* ≤ 0.05). *Post-hoc* analyses demonstrated that protein craving was significantly higher regarding placebo intake compared to protein intake (*p* ≤ 0.01). *Post-hoc* analyses demonstrated that carbohydrate craving was significantly higher regarding placebo intake compared to protein (*p* ≤ 0.001) and carbohydrate (*p* ≤ 0.01) intake. *Post-hoc* analyses demonstrated that vegetable craving was significantly higher regarding placebo intake compared to protein intake (*p* ≤ 0.05). *Post-hoc* analyses demonstrated that fat craving was significantly higher after placebo ingestion compared to protein (time 3: *p* ≤ 0.05; time 4: *p* ≤ 0.05; time 5: *p* ≤ 0.001; time 6: *p* ≤ 0.01; time 7: *p* ≤ 0.001) and fat craving was significantly higher after placebo ingestion compared to fat (time 3: *p* ≤ 0.01; time 4: *p* ≤ 0.05). *Post-hoc* analyses demonstrated that protein craving was significantly higher after placebo ingestion compared to fat (time 3: *p* ≤ 0.01), protein craving was significantly higher after placebo ingestion compared to protein (time 3: *p* ≤ 0.05; time 4: *p* ≤ 0.05; time 5: *p* ≤ 0.01; time 6: *p* ≤ 0.001; time 7: *p* ≤ 0.05) and protein craving was significantly higher after placebo ingestion compared to carbohydrate (time 4: *p* ≤ 0.05). At time 3, *Post-hoc* analyses demonstrated that carbohydrate craving was significantly higher after placebo compared to protein (*p* ≤ 0.05), carbohydrate (*p* ≤ 0.05) and fat ingestion (*p* ≤ 0.05). Furthermore, carbohydrate craving was significantly higher after placebo ingestion compared to protein (time 4: *p* ≤ 0.01; time 5: *p* ≤ 0.001; time 6: *p* ≤ 0.01; time 7: *p* ≤ 0.05) and carbohydrate craving was significantly higher after placebo ingestion compared to carbohydrate (time 4: *p* ≤ 0.05; time 5: *p* ≤ 0.01). At time 5, *Post-hoc* analyses demonstrated that sweets craving was significantly higher after placebo ingestion compared to protein (*p* ≤ 0.05). However, *Post-hoc* analyses demonstrated no significant differences at time 3 and 4.

Isovolumetric-isocaloric conditions: *Post-hoc* analyses demonstrated that protein craving was significantly lower regarding protein intake compared to carbohydrate (*p* ≤ 0.05) and fat (*p* ≤ 0.05) intake. *Post-hoc* analyses demonstrated that vegetable craving was significantly lower regarding protein intake compared to carbohydrate intake (*p* ≤ 0.05). *Post-hoc* analyses demonstrated that fat craving was significantly lower after protein ingestion compared to fat ingestion (time 6: *p* ≤ 0.05) and fat craving was significantly lower after protein ingestion compared to carbohydrate ingestion (time 7: *p* ≤ 0.05). *Post-hoc* analyses demonstrated that protein craving was significantly lower after protein ingestion compared to fat ingestion (time 4: *p* ≤ 0.05; time 5: *p* ≤ 0.05; time 6: *p* ≤ 0.05). Furthermore, carbohydrate craving was significantly lower after protein ingestion compared to fat ingestion (time 6: *p* ≤ 0.05; time 7: *p* ≤ 0.05). At time 5, *Post-hoc* analyses demonstrated that sweets craving was significantly lower after protein ingestion compared to carbohydrate (*p* ≤ 0.01) and fat ingestion (*p* ≤ 0.05).

### Metabolic function

#### Active ghrelin

Placebo: “Time” had no significant effect on active ghrelin levels [*F*_(1, 6)_ = 0.83, *p* = 49].

**Figure 3:**
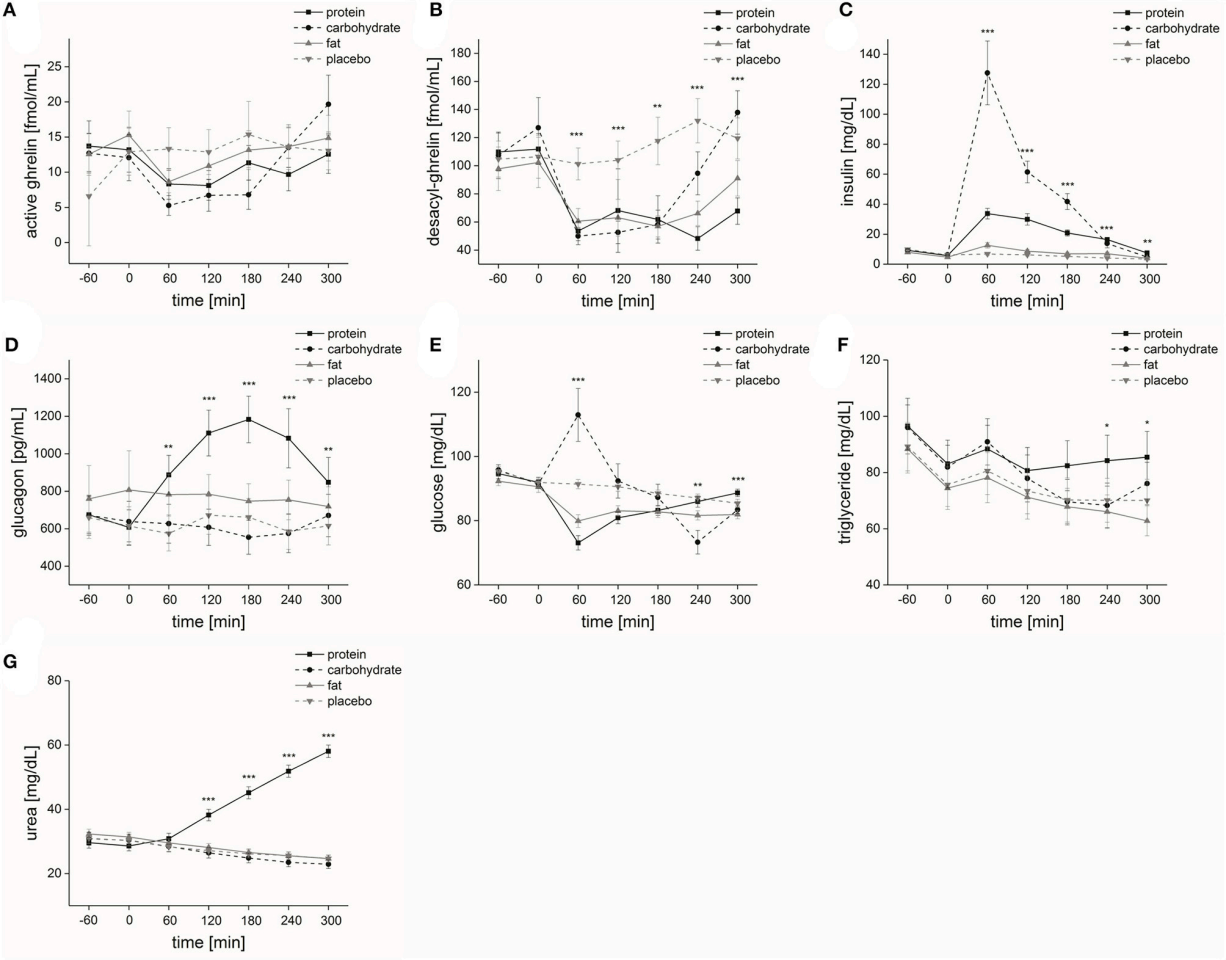
Metabolic parameters. Time course and standard errors of means of the mean metabolic parameters **(A)** active ghrelin, **(B)** desacyl-ghrelin, **(C)** insulin, **(D)** glucagon, **(E)** glucose, **(F)** triglyceride, and **(G)** urea of all participants (*n* = 20) during application of the four different solutions (protein, carbohydrate, fat, or placebo). Statistical significance: ^*^p ≤ 0.05; ^**^p ≤ 0.01; ^***^p ≤ 0.001.

Macronutrient and placebo solution: The factor “time” significantly affected active ghrelin levels [*F*_(1, 6)_ = 6.4, *p* ≤ 0.05]. The factors “solution” and “time × solution” had a non-significant impact on active ghrelin levels [“solution”: *F*_(1, 3)_ = 2.5, *p* = 0.14; “time × solution”: *F*_(3, 6)_ = 3.2, *p* = 0.081] (Figure 3A). Differences in base–to-peak ratios (time 2 compared to time 3–7) for the four different solutions are presented in Table 3. We found a significant positive correlation of active grehlin with hunger and fat, protein and sweets craving for each nutrient solution. Active grehlin significantly correlated with carbohydrate craving for carbohydrate and fat solution and with vegetable craving for fat solution only (for details see Table 4).

**Table 3:** Numerical difference of means of the metabolic parameters.

	**Active ghrelin**		**Desacyl ghrelin**		**Insulin**		**Glucagon**		**Glucose**		**Triglyceride**		**Urea**	
	**Difference**	***p*-value**	**Difference**	***p*-value**	**Difference**	***p*-value**	**Difference**	***p*-value**	**Difference**	***p*-value**	**Difference**	***p*-value**	**Difference**	***p*-value**
	**(fmol/mL)**		**(fmol/mL)**		**(mg/dL)**		**(pg/mL)**		**(mg/dL)**		**(mg/dL)**		**(mg/dL)**	
**PROTEIN SOLUTION**														
m3-m2	−7.7	**≤0.001**	−58.3	**≤0.001**	28.0	**≤0.001**	280.0	**≤0.001**	−18.6	**≤0.001**	5.3	**≤0.05**	2.3	**≤0.001**
m4-m2	−7.6	**≤0.01**	−43.7	0.17	24.2	**≤0.001**	503.1	**≤0.001**	−11.1	**≤0.001**	−2.4	0.32	9.6	**≤0.001**
m5-m2	−6.7	**≤0.05**	−50.1	**≤0.01**	15.1	**≤0.001**	575.7	**≤0.001**	−8.6	**≤0.001**	−0.65	0.80	16.6	**≤0.001**
m6-m2	−4.8	**≤0.05**	−63.6	**≤0.001**	10.7	**≤0.001**	475.5	**≤0.001**	−6.0	**≤0.001**	1.2	0.68	23.3	**≤0.001**
m7-m2	−1.6	0.43	−42.3	**≤0.001**	1.8	0.071	240.4	0.012	−3.3	**≤0.05**	2.4	0.48	29.5	**≤0.001**
**CARBO-HYDRATE SOLUTION**														
m3-m2	−8.4	**≤0.01**	−91.4	**≤0.001**	121.4	**≤0.001**	−11.0	0.86	21.5	**≤0.05**	9.0	**≤0.01**	−1.9	**≤0.001**
m4-m2	−6.8	**≤0.05**	−86.6	**≤0.001**	55.4	**≤0.001**	−30.7	0.57	0.95	0.86	−4.0	0.13	−3.9	**≤0.001**
m5-m2	−5.0	**≤0.05**	−72.6	**≤0.001**	35.5	**≤0.001**	−83.6	0.26	−4.2	0.32	−12.3	**≤0.001**	−5.5	**≤0.001**
m6-m2	2.3	0.35	−37.2	0.08	7.6	**≤0.05**	−63.3	0.40	−18.1	**≤0.001**	−13.6	**≤0.01**	−6.9	**≤0.001**
m7-m2	8.4	**≤0.001**	10.8	0.59	−1.5	0.12	32.4	0.79	−8.0	**≤0.001**	−5.9	0.14	−7.5	**≤0.001**
**FAT SOLUTION**														
m3-m2	−8.3	**≤0.01**	−50.4	**≤0.001**	7.8	**≤0.001**	−24.6	0.89	−10.6	**≤0.001**	3.8	0.17	−1.9	**≤0.001**
m4-m2	−6.5	0.06	−44.0	**≤0.01**	4.0	**≤0.001**	−22.9	0.90	−7.5	**≤0.001**	−3.2	0.08	−3.3	**≤0.001**
m5-m2	−2.6	0.17	−50.1	**≤0.01**	2.2	**≤0.01**	−59.8	0.73	−7.8	**≤0.001**	−6.6	**≤0.05**	−4.9	**≤0.001**
m6-m2	−2.0	0.18	−44.7	**≤0.01**	2.3	**≤0.05**	−53.5	0.77	−8.9	**≤0.001**	−8.4	**≤0.05**	−5.9	**≤0.001**
m7-m2	0.29	0.87	−20.3	0.18	−0.77	0.068	−88.4	0.55	−8.7	**≤0.001**	−11.6	**≤0.01**	−6.8	**≤0.001**
**PLACEBO SOLUTION**														
m3-m2	0.43	0.72	−5.1	0.47	0.63	0.12	40.0	0.41	−0.53	0.61	4.9	**≤0.01**	−1.9	**≤0.001**
m4-m2	0.67	0.66	−2.5	0.81	0.05	0.92	57.4	0.15	−1.3	0.16	−2.2	0.22	−3.1	**≤0.001**
m5-m2	1.8	0.33	11.2	0.35	−1.0	0.10	47.4	0.33	−3.4	0.10	−5.4	**≤0.05**	−4.1	**≤0.001**
m6-m2	0.7	0.64	25.6	0.054	−2.1	**≤0.001**	−28.6	0.53	−4.8	**≤0.01**	−5.5	**≤0.05**	−4.7	**≤0.001**
m7-m2	0.024	0.99	13.0	0.18	−2.7	**≤0.001**	0.66	0.99	−6.5	**≤0.01**	−5.6	0.11	−5.6	**≤0.001**

*m, mean of session; significant differences are presented in bold*.

**Table 4:** Correlation of the psychophysical parameters (hunger, food craving) with active ghrelin and desacyl ghrelin.

	**Hunger**		**Fat craving**		**Protein craving**		**Carbohydrate craving**		**Sweets craving**		**Vegetable craving**	
	***r***	***p*-value**	***r***	***p*-value**	***r***	***p*-value**	***r***	***p*-value**	***r***	***p*-value**	***r***	***p*-value**
**ACTIVE GHRELIN**												
Protein solution	0.87	**≤0.05**	0.90	**≤0.01**	0.82	**≤0.05**	0.65	0.12	0.78	**≤0.05**	0.70	0.082
Carbohydrate solution	0.94	**≤0.001**	0.91	**≤0.01**	0.87	**≤0.05**	0.79	**≤0.05**	0.89	**≤0.01**	0.68	0.091
Fat solution	0.97	**≤0.001**	0.97	**≤0.001**	0.92	**≤0.01**	0.87	**≤0.05**	0.95	**≤0.001**	0.89	**≤0.01**
Placebo solution	0.18	0.70	0.15	0.75	0.17	0.72	0.31	0.50	0.28	0.55	0.29	0.53
**DESACYL GHRELIN**												
Protein solution	0.61	0.15	0.73	0.060	0.49	0.27	0.27	0.57	0.50	0.25	0.28	0.54
Carbohydrate solution	0.92	**≤0.01**	0.90	**≤0.01**	0.73	0.064	0.66	0.11	0.77	**≤0.05**	0.48	0.28
Fat solution	0.62	0.14	0.69	0.086	0.41	0.36	0.28	0.54	0.56	0.19	0.34	0.45
Placebo solution	0.82	**≤0.05**	0.81	**≤0.05**	0.82	**≤0.05**	0.84	**≤0.05**	0.84	**≤0.05**	0.86	**≤0.05**

*r, Pearson correlation coefficient. Significant differences are presented in bold*.

#### Desacyl ghrelin

Placebo: “Time” significantly affected desacyl ghrelin levels [*F*_(1, 6)_ = 3.1, *p* ≤ 0.05]. However, *Post-hoc* analyses demonstrated no significant differences.

Macronutrient and placebo solution: The factors “time,” “solution,” and “time × solution” had a significant impact on desacyl ghrelin levels [“time”: *F*_(1, 6)_ = 17.3, *p* ≤ 0.001; “solution”: *F*_(1, 3)_ = 8.5, *p* ≤ 0.01; “time × solution”: *F*_(3, 6)_ = 5.2, *p* ≤ 0.001]. The comparison of desacyl ghrelin levels at each measurement point showed that at time 3 [*F*_(1, 3)_ = 20.0, *p* ≤ 0.001], time 4 [*F*_(1, 3)_ = 13.1, *p* ≤ 0.001], time 5 [*F*_(1, 3)_ = 6.5, *p* ≤ 0.01], time 6 [*F*_(1, 3)_ = 18.9, *p* ≤ 0.001] and time 7 [*F*_(1, 3)_ = 11.0, *p* ≤ 0.001] desacyl ghrelin levels significantly differed between the four solutions (Figure 3B). Differences in base-to-peak ratios (time 2 compared to time 3–7) for the four different solutions are presented in Table 3. For the results of the Pearson correlation of desacyl ghrelin with hunger and food craving calculated for each nutrient solution separately see Table 4.

Isovolumetric conditions: *Post-hoc* analyses demonstrated that desacyl ghrelin levels were significantly higher regarding placebo intake compared to protein intake (*p* ≤ 0.001). At time 3, *Post-hoc* analyses demonstrated that desacyl ghrelin levels were significantly higher after placebo compared to protein (*p* ≤ 0.001), carbohydrate (*p* ≤ 0.001) and fat intake (*p* ≤ 0.001). At time 4, *Post-hoc* analyses demonstrated that desacyl ghrelin levels were significantly higher after placebo compared to protein (*p* ≤ 0.001), carbohydrate (*p* ≤ 0.01) and fat intake (*p* ≤ 0.05). At time 5, *Post-hoc* analyses demonstrated that desacyl ghrelin levels were significantly higher after placebo compared to carbohydrate (*p* ≤ 0.01) and fat intake (*p* ≤ 0.01). At time 6, *Post-hoc* analyses demonstrated that desacyl ghrelin levels were significantly higher after placebo compared to protein (*p* ≤ 0.001), carbohydrate (*p* ≤ 0.05) and fat intake (*p* ≤ 0.001). At time 7, *Post-hoc* analyses demonstrated that desacyl ghrelin levels were significantly higher after placebo compared to protein intake (*p* ≤ 0.01).

Isovolumetric-isocaloric conditions: *Post-hoc* analyses demonstrated that desacyl ghrelin levels were significantly lower regarding protein intake compared to carbohydrate intake (*p* ≤ 0.05). At time 6, *Post-hoc* analyses demonstrated that desacyl ghrelin levels were significantly lower after protein intake compared to carbohydrate intake (*p* ≤ 0.05). At time 7, *Post-hoc* analyses demonstrated that desacyl ghrelin levels were significantly lower after protein compared to carbohydrate intake (*p* ≤ 0.01) and desacyl ghrelin levels were significantly higher after carbohydrate intake compared to fat intake (*p* ≤ 0.05).

#### Insulin

Placebo: “Time” had a significant impact on insulin levels [*F*_(1, 6)_ = 25.2, *p* ≤ 0.001]. *Post-hoc* analyses demonstrated that insulin levels were significantly higher at time 2 compared to time 6 (*p* ≤ 0.001) and time 7 (*p* ≤ 0.05).

Macronutrient and placebo solution: The factors “time”, “solution” and “time × solution” had a significant impact on insulin levels [“time”: *F*_(1, 6)_ = 37.3, *p* ≤ 0.001; “solution”: *F*_(1, 3)_ = 51.2, *p* ≤ 0.001; “time × solution”: *F*_(3, 6)_ = 26.0, *p* ≤ 0.001]. The comparison of insulin levels at each measurement point showed that at time 3 [*F*_(1, 3)_ = 30.5, *p* ≤ 0.001], time 4 [*F*_(1, 3)_ = 41.0, *p* ≤ 0.001], time 5 [*F*_(1, 3)_ = 41.1, *p* ≤ 0.001], time 6 [*F*_(1, 3)_ = 12.3, *p* ≤ 0.001], and time 7 [*F*_(1, 3)_ = 6.9, *p* ≤ 0.01] insulin levels significantly differed between the four solutions (Figure 3C). Differences in base to peaks ratios (time 2 compared to time 3–7) for the four different solutions are presented in Table 3.

Isovolumetric conditions: *Post-hoc* analyses demonstrated that insulin levels were significantly lower regarding placebo intake compared to carbohydrate (*p* ≤ 0.001) and protein (*p* ≤ 0.001) intake. At time 3, *Post-hoc* analyses demonstrated that insulin levels were significantly lower after placebo intake compared to carbohydrate (*p* ≤ 0.001), protein (*p* ≤ 0.001) and fat (*p* ≤ 0.05) intake. At time 4, *Post-hoc* analyses demonstrated that insulin levels were significantly lower after placebo intake compared to carbohydrate (*p* ≤ 0.001) and protein (*p* ≤ 0.001) intake. At time 5, *Post-hoc* analyses demonstrated that insulin levels were significantly lower after placebo intake compared to carbohydrate (*p* ≤ 0.001) and protein (*p* ≤ 0.001) intake. At time 6, *Post-hoc* analyses demonstrated that insulin levels were significantly lower after placebo intake compared to protein (*p* ≤ 0.001) and carbohydrate (*p* ≤ 0.05) intake. At time 7, *Post-hoc* analyses demonstrated that insulin levels were significantly lower after placebo intake compared to protein intake (*p* ≤ 0.05).

Isovolumetric-isocaloric conditions: *Post-hoc* analyses demonstrated that insulin levels were significantly higher regarding carbohydrate intake compared to protein (*p* ≤ 0.001) and fat (*p* ≤ 0.001) intake and significantly higher regarding protein intake compared to fat intake (*p* ≤ 0.001). At time 3, *Post-hoc* analyses demonstrated that insulin levels were significantly higher after carbohydrate intake compared to protein (*p* ≤ 0.01) and fat (*p* ≤ 0.001) intake and insulin levels were significantly higher after protein intake compared to fat intake (*p* ≤ 0.001). At time 4, *Post-hoc* analyses demonstrated that insulin levels were significantly higher after carbohydrate intake compared to protein (*p* ≤ 0.01) and fat (*p* ≤ 0.001) intake and insulin levels were significantly higher after protein intake compared to fat intake (*p* ≤ 0.001). At time 5, *Post-hoc* analyses demonstrated that insulin levels were significantly higher after carbohydrate intake compared to protein (*p* ≤ 0.01) and fat (*p* ≤ 0.001) intake and insulin levels were significantly higher after protein intake compared to fat intake (*p* ≤ 0.001). At time 6, *Post-hoc* analyses demonstrated that insulin levels were significantly higher after protein intake compared to fat intake (*p* ≤ 0.001). At time 7, *Post-hoc* analyses demonstrated that insulin levels were significantly higher after protein intake compared to fat intake (*p* ≤ 0.05).

#### Glucagon

Placebo: “Time” had no significant impact on glucagon levels [*F*_(1, 6)_ = 1.6, *p* = 0.18].

Macronutrient and placebo solution: The factor “time” had no significant effect on glucagon levels [*F*_(1, 6)_ = 2.1, *p* = 0.12]. The factors “solution” and “time × solution” had a significant impact on glucagon levels [“solution”: *F*_(1, 3)_ = 13.3, *p* ≤ 0.001; “time × solution”: *F*_(3, 6)_ = 4.4, *p* ≤ 0.01]. The comparison of glucagon levels at each measurement point showed that at time 3 [*F*_(1, 3)_ = 8.8, *p* ≤ 0.001], time 4 [*F*_(1, 3)_ = 11.6, *p* ≤ 0.001], time 5 [*F*_(1, 3)_ = 22.7, *p* ≤ 0.001], time 6 [*F*_(1, 3)_ = 10.8, *p* ≤ 0.001] and time 7 [*F*_(1, 3)_ = 5.5, *p* ≤ 0.01] glucagon levels significantly differed between the four solutions (Figure 3D). Differences in base-to-peak ratios (time 2 compared to time 3–7) for the four different solutions are presented in Table 3.

Isovolumetric conditions: *Post-hoc* analyses demonstrated that glucagon levels were significantly lower regarding placebo intake compared to protein (*p* ≤ 0.001) and fat (*p* ≤ 0.05) intake. At time 3, *Post-hoc* analyses demonstrated that glucagon levels were significantly lower after placebo intake compared to protein intake (*p* ≤ 0.001). At time 4, *Post-hoc* analyses demonstrated that glucagon levels were significantly lower after placebo intake compared to protein intake (*p* ≤ 0.001). At time 5, *Post-hoc* analyses demonstrated that glucagon levels were significantly lower after placebo intake compared to protein intake (*p* ≤ 0.001). At time 6, *Post-hoc* analyses demonstrated that glucagon levels were significantly lower after placebo intake compared to protein intake (*p* ≤ 0.01). At time 7, *Post-hoc* analyses demonstrated that glucagon levels were significantly lower after placebo intake compared to protein intake (*p* ≤ 0.05).

Isovolumetric-isocaloric conditions: *Post-hoc* analyses demonstrated that glucagon levels were significantly higher regarding protein intake compared to carbohydrate intake (*p* ≤ 0.01). At time 3, *Post-hoc* analyses demonstrated that glucagon levels were significantly higher after protein intake compared to carbohydrate intake (*p* ≤ 0.05) and glucagon levels were significantly lower after carbohydrate intake compared to fat intake (*p* ≤ 0.01). At time 4, *Post-hoc* analyses demonstrated that glucagon levels were significantly higher after protein intake compared to carbohydrate (*p* ≤ 0.01) and fat (*p* ≤ 0.05) intake. At time 5, *Post-hoc* analyses demonstrated that glucagon levels were significantly higher after protein intake compared to carbohydrate (*p* ≤ 0.001) and fat (*p* ≤ 0.001) intake. At time 6, *Post-hoc* analyses demonstrated that glucagon levels were significantly higher after protein intake compared to carbohydrate intake (*p* ≤ 0.01). At time 7, *Post-hoc* analyses demonstrated that glucagon levels were significantly higher after protein intake compared to carbohydrate intake (*p* ≤ 0.05).

#### Glucose

Placebo: “Time” had a significant impact on glucose levels [*F*_(1, 6)_ = 18.1, *p* ≤ 0.001]. *Post-hoc* analyses demonstrated that glucose levels were significantly higher at time 2 compared to time 7 (*p* ≤ 0.05).

Macronutrient and placebo solution: The factors “time,” “solution,” and “time × solution” had a significant impact on glucose levels [“time”: *F*_(1, 6)_ = 11.0, *p* ≤ 0.001; “solution”: *F*_(1, 3)_ = 6.5, *p* ≤ 0.01; “time × solution”: *F*_(3, 6)_ = 13.7, *p* ≤ 0.001]. The comparison of glucose levels at each measurement point showed that at time 3 [*F*_(1, 3)_ = 18.6, *p* ≤ 0.001], time 6 [*F*_(1, 3)_ = 9.1, *p* ≤ 0.01], and time 7 [*F*_(1, 3)_ = 9.0, *p* ≤ 0.001] glucose levels significantly differed between the four solutions (Figure 3E). Differences in base-to-peak ratios (time 2 compared to time 3–7) for the four different solutions are presented in Table 3. Isovolumetric conditions: *Post-hoc* analyses demonstrated that glucose levels were significantly higher regarding placebo compared to protein (*p* ≤ 0.01) and fat (*p* ≤ 0.001) intake. At time 3, *Post-hoc* analyses demonstrated that glucose levels were significantly higher after placebo intake compared to protein (*p* ≤ 0.001) and fat (*p* ≤ 0.001) intake. At time 6, *Post-hoc* analyses demonstrated that glucose levels were significantly higher after placebo intake compared to carbohydrate (*p* ≤ 0.05) and fat intake (*p* ≤ 0.01).

Isovolumetric-isocaloric conditions: At time 3, *Post-hoc* analyses demonstrated that glucose levels were significantly higher after carbohydrate intake compared to protein (*p* ≤ 0.001) and fat intake (*p* ≤ 0.01). At time 6, *Post-hoc* analyses demonstrated that glucose levels were significantly lower after carbohydrate intake compared to protein intake (*p* ≤ 0.01) and glucose levels were significantly lower after fat intake compared to protein intake (*p* ≤ 0.05). At time 7, *Post-hoc* analyses demonstrated that glucose levels were significantly higher after protein intake compared to carbohydrate (*p* ≤ 0.05) and fat intake (*p* ≤ 0.001).

#### Triglycerides

Placebo: Time” had a significant impact on triglyceride levels [*F*_(1, 6)_ = 13.8, *p* ≤ 0.001]. *Post-hoc* analyses did not show significant differences between session 2, 0 min and session 3–7, 60–300 min.

Macronutrient and placebo solution: The factor “solution” had no significant effect on triglyceride levels [*F*_(1, 3)_ = 1.5, *p* = 0.24]. The factors “time” and “time × solution” had a significant impact on triglyceride levels [“time”: *F*_(1, 6)_ = 20.3, *p* ≤ 0.001; “time × solution”: *F*_(3, 6)_ = 2.8, *p* ≤ 0.05]. The comparison of triglyceride levels at each measurement point showed that at time 6 [*F*_(1, 3)_ = 3.5, *p* ≤ 0.05] and time 7 [*F*_(1, 3)_ = 4.5, *p* ≤ 0.05] triglyceride levels significantly differed between the four solutions (Figure 3F). Differences in base-to-peak ratios (time 2 compared to time 3–7) for normal rate of intake are presented in Table 3.

Isovolumetric conditions: *Post-hoc* analyses demonstrated no significant differences at time 7.

Isovolumetric-isocaloric conditions: At time 6, *Post-hoc* analyses demonstrated that triglyceride levels were significantly higher after protein intake compared to carbohydrate intake. (*p* ≤ 0.01).

#### Urea

Placebo: “Time” had a significant impact on urea levels [*F*_(1, 6)_ = 93.2, *p* ≤ 0.001]. *Post-hoc* analyses demonstrated that urea levels were significantly higher at time 2 compared to time 3 (*p* ≤ 0.001), time 4 (*p* ≤ 0.001), time 5 (*p* ≤ 0.001), time 6 (*p* ≤ 0.001) and time 7 (*p* ≤ 0.001).

Macronutrient and placebo solution: The factors “time”, “solution” and “time × solution” had a significant impact on urea levels [“time”: *F*_(1, 6)_ = 22.0, *p* ≤ 0.001; “solution”: *F*_(1, 3)_ = 43.6, *p* ≤ 0.001; “time × solution”: *F*_(3, 6)_ = 357.3, *p* ≤ 0.001]. The comparison of urea levels at each measurement point showed that at time 4 [*F*_(1, 3)_ = 27.0, *p* ≤ 0.001], time 5 [*F*_(1, 3)_ = 79.5, *p* ≤ 0.001], time 6 [*F*_(1, 3)_ = 172.5, *p* ≤ 0.001] and time 7 [*F*_(1, 3)_ = 348.3, *p* ≤ 0.001] urea levels significantly differed between the four solutions (Figure 3G). Differences in base- to-peak ratios (time 2 compared to time 3–7) for the four different solutions are presented in Table 3.

Isovolumetric conditions: *Post-hoc* analyses demonstrated that urea levels were significantly lower regarding placebo intake compared to protein intake (*p* ≤ 0.001). At time 4–7, *Post-hoc* analyses demonstrated that urea levels were significantly lower after placebo intake compared to protein intake (time 4: *p* ≤ 0.001; time 5: *p* ≤ 0.001; time 6: *p* ≤ 0.001; time 7: *p* ≤ 0.001).

Isovolumetric-isocaloric conditions: *Post-hoc* analyses demonstrated that urea levels were significantly higher regarding protein intake compared to carbohydrate (*p* ≤ 0.001) and fat (*p* ≤ 0.001) intake. At time 4–7, *Post-hoc* analyses demonstrated that urea levels were significantly higher after protein intake compared to carbohydrate (time 4: *p* ≤ 0.001; time 5: *p* ≤ 0.001; time 6: *p* ≤ 0.001; time 7: *p* ≤ 0.001) and fat intake (time 4: *p* ≤ 0.001; time 5: *p* ≤ 0.001; time 6: *p* ≤ 0.001; time 7: *p* ≤ 0.001).

### Cognitive function

#### Alertness

Placebo: “Time” had no significant impact on alertness [*F*_(1, 6)_ = 0.48, *p* = 0.65].

**Figure 4:**
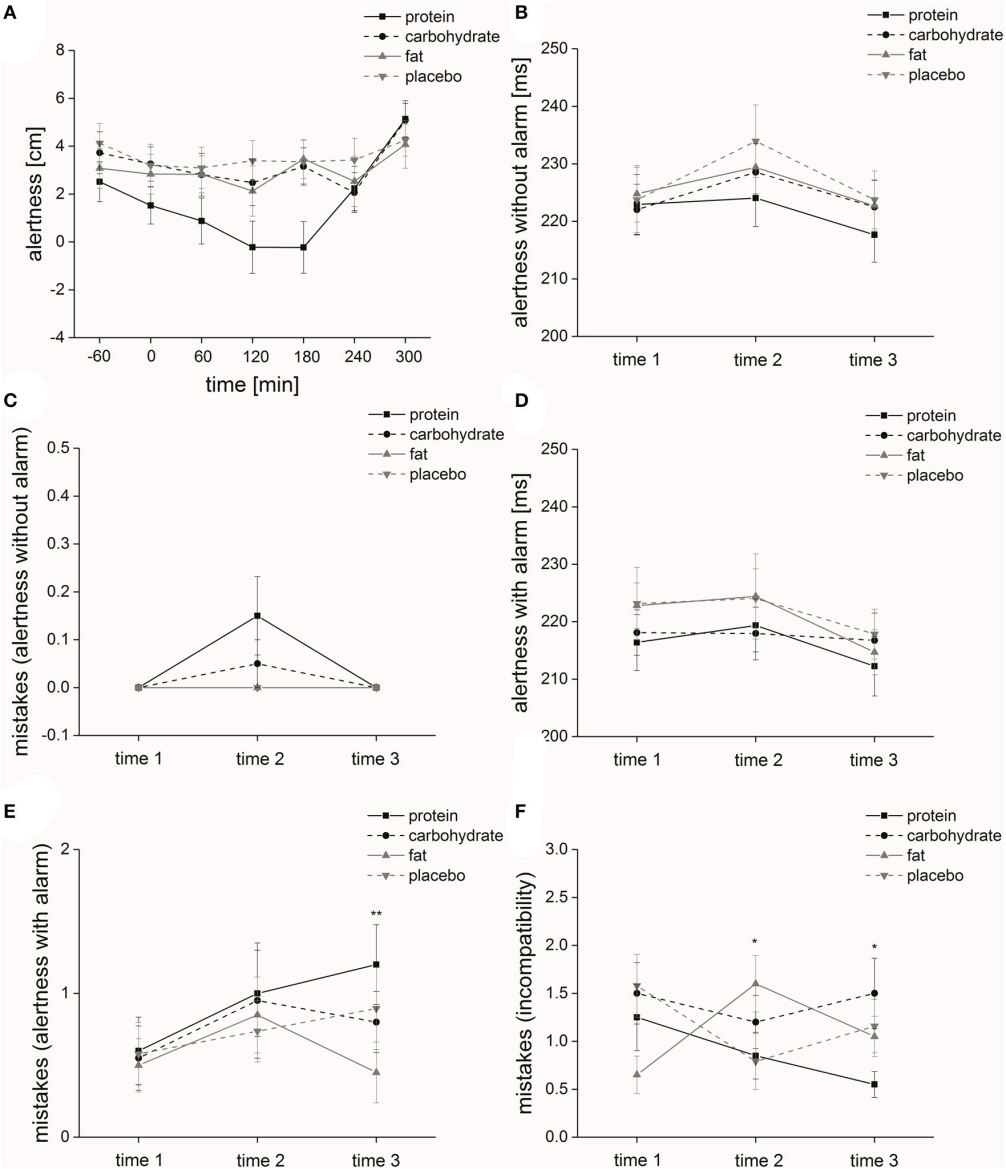
Cognitive function. Time course and standard errors of means of the mean cognitive function parameters **(A)** alertness, **(B)** alertness without alarm, **(C)** mistakes (alertness without alarm), **(D)** alertness with alarm, **(E)** mistakes (alertness with alarm) and **(F)** mistakes (incompatibility) of all participants (*n* = 20) during application of the four different solutions (protein, carbohydrate, fat, or placebo). Statistical significance: ^*^p ≤ 0.05; ^**^p ≤ 0.01.

Macronutrient and placebo solution: “Time × solution” did not significantly affect alertness [*F*_(3, 6)_ = 1.5, *p* = 0.18]. The factors “time” and “solution” had a significant impact on alertness [“time”: *F*_(1, 6)_ = 3.2, *p* ≤ 0.05; “solution”: *F*_(1, 3)_ = 4.7, *p* ≤ 0.01] (Figure 4A).

Isovolumetric conditions: *Post-hoc* analyses demonstrated that alertness ratings were significantly higher regarding placebo intake compared to protein intake (*p* ≤ 0.05).

Isovolumetric-isocaloric conditions: *Post-hoc* analyses demonstrated that alertness ratings were significantly lower regarding protein intake compared to carbohydrate intake (*p* ≤ 0.01).

#### Alertness without acoustic signal

Placebo: In terms of reaction time, we found a significant effect of the factor “time” [*F*_(1, 2)_ = 8.9, *p* ≤ 0.001]. *Post-hoc* analyses showed that reaction times at time 2 were significantly higher compared to time 1 (*p* ≤ 0.01) and time 3 (*p* ≤ 0.05). Regarding error rate, the factor “time” had no significant impact (no error occurred).

Macronutrient and placebo solution: We found a significant effect of the factor “time” [*F*_(1, 2)_ = 10.6, *p* ≤ 0.001] (Figure 4B), but no significant effects of the factors “solution” and “time × solution” on the parameter “alertness without acoustic signal” [“solution”: *F*_(1, 3)_ = 1.4, *p* = 0.25; “time × solution”: *F*_(2, 3)_ = 1.5, *p* = 0.21] on reaction time. Regarding error rate, the factor “time” [$\chi_{\left(2\right)}^{2}$ = 8.0, *p* = 0.018] had a significant impact (Figure 4C), but “solution” and “time × solution” did not affect error rate [“solution”: $\chi_{\left(3\right)}^{2}$ = 3.7, *p* = 0.30; “time × solution”: $\chi_{\left(11\right)}^{2}$ = 17.0, *p* = 0.11] (Table 5).

**Table 5:** Cognitive parameters presented as means including standard error of means (SEM).

	**Alertness without acoustic signal**	**Alertness with acoustic signal**	**Working memory**	**Incompatibility**
Protein solution RT^*^ [ms]	Test 1: 222.9 ± 5.2	Test 1: 216.4 ± 4.9	Test 1: 626.4 ± 36.5	Test 1: 399.3 ± 9.8
	Test 2: 224.1 ± 4.9	Test 2: 219.4 ± 4.6	Test 2: 630.9 ± 44.8	Test 2: 395.4 ± 11.2
	Test 3: 217.7 ± 4.7	Test 3: 212.3 ± 5.2	Test 3: 581.6 ± 35.0	Test 3: 381.6 ± 7.5
Protein solution mistakes	Test 1: 0 ± 0	Test 1: 0.60 ± 0.23	Test 1: 1.1 ± 0.23	Test 1: 1.3 ± 0.35
	Test 2: 0.15 ± 0.082	Test 2: 1.0 ± 0.30	Test 2: 1.6 ± 0.31	Test 2: 0.85 ± 0.24
	Test 3: 0 ± 0	Test 3: 1.2 ± 0.28	Test 3: 1.1 ± 0.31	Test 3: 0.55 ± 0.14
Carbohydrate solution RT^*^ [ms]	Test 1: 225.1 ± 4.4	Test 1: 218.1 ± 3.9	Test 1: 634.4 ± 37.6	Test 1: 384.4 ± 12.1
	Test 2: 228.6 ± 5.0	Test 2: 218.0 ± 4.6	Test 2: 650.2 ± 44.1	Test 2: 385.9 ± 12.5
	Test 3: 222.5 ± 4.7	Test 3: 216.8 ± 4.8	Test 3: 630.2 ± 44.9	Test 3: 394.6 ± 13.5
Carbohydrate solution mistakes	Test 1: 0 ± 0	Test 1: 0.55 ± 0.22	Test 1: 0.80 ± 0.27	Test 1: 1.5 ± 0.32
	Test 2: 0.05 ± 0.05	Test 2: 0.95 ± 0.40	Test 2: 1.7 ± 0.44	Test 2: 1.2 ± 0.28
	Test 3: 0 ± 0	Test 3: 0.80 ± 0.21	Test 3: 1.1 ± 0.33	Test 3: 1.5 ± 0.37
Fat solution RT^*^ [ms]	Test 1: 224.8 ± 4.9	Test 1: 222.8 ± 3.9	Test 1: 619.1 ± 37.7	Test 1: 388.8 ± 8.2
	Test 2: 229.4 ± 4.6	Test 2: 224.4 ± 7.4	Test 2: 598.3 ± 35.8	Test 2: 394.9 ± 8.6
	Test 3: 222.7 ± 4.4	Test 3: 214.7 ± 3.9	Test 3: 581.8 ± 32.8	Test 3: 382.0 ± 7.3
Fat solution mistakes	Test 1: 0 ± 0	Test 1: 0.50 ± 0.18	Test 1: 1.3 ± 0.44	Test 1: 0.65 ± 0.2
	Test 2: 0 ± 0	Test 2: 0.85 ± 0.26	Test 2: 1.3 ± 0.38	Test 2: 1.6 ± 0.29
	Test 3: 0 ± 0	Test 3: 0.45 ± 0.21	Test 3: 1.1 ± 0.44	Test 3: 1.1 ± 0.21
Placebo solution RT^*^ [ms]	Test 1: 223.7 ± 5.7	Test 1: 223.2 ± 6.3	Test 1: 566.8 ± 32.2	Test 1: 393.3 ± 8.8
	Test 2: 233.9 ± 6.3	Test 2: 224.1 ± 5.2	Test 2: 574.7 ± 30.9	Test 2: 399.2 ± 9.1
	Test 3: 223.7 ± 5.0	Test 3: 217.8 ± 4.3	Test 3: 574.6 ± 30.3	Test 3: 384.9 ± 8.2
Placebo solution mistakes	Test 1: 0 ± 0	Test 1: 0.58 ± 0.22	Test 1: 1.6 ± 0.44	Test 1: 1.6 ± 0.33
	Test 2: 0 ± 0	Test 2: 0.74 ± 0.21	Test 2: 0.84 ± 0.24	Test 2: 0.79 ± 0.29
	Test 3: 0 ± 0	Test 3: 0.89 ± 0.29	Test 3: 0.89 ± 0.23	Test 3: 1.2 ± 0.28

* *RT, reaction time*.

#### Alertness with acoustic signal

Placebo: In terms of reaction time, we found no significant effect of the factor “time” [*F*_(1, 2)_ = 2.4, *p* = 0.12]. Regarding error rate, the factor “time” had no significant impact [$\chi_{\left(2\right)}^{2}$ = 2.8, *p* = 0.25].

Macronutrient and placebo solution: In terms of reaction time, we found a significant effect of the factor “time” [*F*_(1, 2)_ = 5.9, *p* ≤ 0.01] (Figure 4D), but no significant effect of the factors “solution” and “time × solution” on the parameter “alertness with acoustic signal” [“solution”: *F*_(1, 3)_ = 2.1, *p* = 0.12; “time × solution”: *F*_(2, 3)_ = 0.76, *p* = 0.52]. The factor “solution” had no significant effect on error rate [$\chi_{\left(3\right)}^{2}$ = 4.8, *p* = 0.19]. However, the factors “time” [$\chi_{\left(2\right)}^{2}$ = 8.4, *p* ≤ 0.05] and “time × solution” [$\chi_{\left(11\right)}^{2}$ = 21.7, *p* ≤ 0.05] significantly affected error rate (Table 5).

Isovolumetric-isocaloric conditions: *Post-hoc* analyses demonstrated that at time 3 the error rate was significantly higher after protein ingestion compared to fat ingestion (*p* ≤ 0.01) (Figure 4E).

#### Working memory

Placebo: In terms of reaction time, we found no significant effect of the factor “time” [*F*_(1, 2)_ = 0.10, *p* = 0.88]. Regarding error rate, the factor “time” had no significant impact [$\chi_{\left(2\right)}^{2}$ = 2.5, *p* = 0.29].

Macronutrient and placebo solution: We found no significant effect of the factors “time”, “solution” and “time × solution” on reaction time [“time”: *F*_(1, 2)_ = 3.0, *p* = 0.078; “solution”: *F*_(1, 3)_ = 2.8, *p* = 0.062; “time × solution”: *F*_(2, 3)_ = 0.75, *p* = 0.55] (Table 5). The factors “time” [$\chi_{\left(2\right)}^{2}$ = 5.4, *p* = 0.066], “solution” [$\chi_{\left(3\right)}^{2}$ = 0.99, *p* = 0.81] and “time × solution” [$\chi_{\left(11\right)}^{2}$ = 10.9, *p* = 0.45] had no significant effect on error rate (Table 5).

#### Incompatibility

Placebo: In terms of reaction time, we found a significant effect of the factor “time” [*F*_(1, 2)_ = 6.1, *p* ≤ 0.01]. *Post-hoc* analyses showed that reaction times at time 2 were significantly higher compared to time 3 (*p* ≤ 0.01). Regarding error rate, the factor “time” had no significant impact [$\chi_{\left(2\right)}^{2}$ = 4.6, *p* = 0.10].

Macronutrient and placebo solution: Reaction time was not affected in terms of the factors “time,” “solution” and “time × solution” [“time”: *F*_(1, 2)_ = 1.7, *p* = 0.20; “solution”: *F*_(1, 3)_ = 1.3, *p* = 0.27; “time × solution”: *F*_(2, 3)_ = 2.5, *p* = 0.065]. The factors “time” and “solution” had no significant effect on error rate [“time”: $\chi_{\left(2\right)}^{2}$ = 0.42, *p* = 0.81; “solution”: $\chi_{\left(3\right)}^{2}$ = 6.7, *p* = 0.081], but “time × solution” [$\chi_{\left(11\right)}^{2}$ = 25.6, *p* ≤ 0.01] significantly affected error rate (Table 5).

Isovolumetric conditions: *Post-hoc* analyses demonstrated that at time 3 the error rate was significantly higher after placebo intake compared to protein intake (*p* ≤ 0.05) (Figure 4F).

Isovolumetric-isocaloric conditions: *Post-hoc* analyses demonstrated that at time 2 and 3 the error rate was significantly lower after protein ingestion compared to other solutions (*time 2:* fat solution: *p* ≤ 0.05; time 3: carbohydrate solution: *p* ≤ 0.05) (Figure 4F).

### Olfactory parameters

#### Threshold

Placebo: The factor “time” [$\chi_{\left(2\right)}^{2}$ = 3.1, *p* = 0.21] had no significant effect on subjects' *n*-butanol threshold.

**Figure 5:**
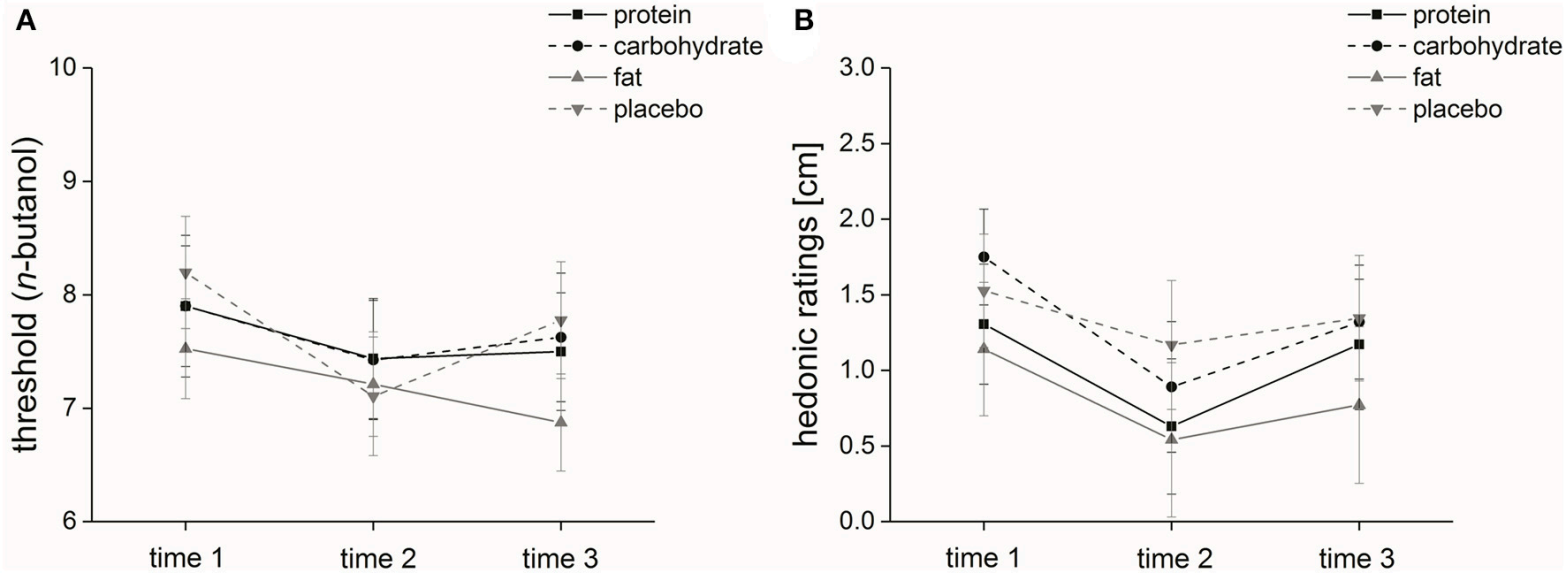
Olfactory parameters. Time course and standard error of means of the mean olfactory parameters **(A)** threshold (*n*-butanol) and **(B)** hedonic ratings.

Macronutrient and placebo solution: The factor “time” [$\chi_{\left(2\right)}^{2}$ = 8.9, *p* ≤ 0.05] significantly influenced subjects' *n*-butanol threshold (Figure 5A), but “solution” and “time × solution” had no significant influence on the *n*-butanol thresholds [“solution”: $\chi_{\left(3\right)}^{2}$ = 3.8, *p* = 0.28; “time × solution”: $\chi_{\left(11\right)}^{2}$ = 13.5, *p* = 0.26; threshold scores; Table 6].

**Table 6:** Olfactory parameters presented as means including standard error of means (SEM).

	**Threshold**	**Discrimination rate**	**Identification rate**	**Intensity rating**	**Hedonic rating**
Protein solution	Test 1: 7.9 ± 0.62	Test 1: 12.0 ± 0.51	Test 1: 13.4 ± 0.367	Test 1: 13.5 ± 0.45	Test 1: 1.3 ± 0.40
	Test 2: 7.4 ± 0.53	Test 2: 12.0 ± 0.60	Test 2: 13.5 ± 0.34	Test 2: 13.7 ± 0.47	Test 2: 0.63 ± 0.45
	Test 3: 7.5 ± 0.52	Test 3: 12.0 ± 0.54	Test 3: 13.3 ± 0.42	Test 3: 13.8 ± 0.50	Test 3: 1.2 ± 0.43
Carbohydrate solution	Test 1: 7.9 ± 0.53	Test 1: 12.1 ± 0.64	Test 1: 13.4 ± 0.37	Test 1: 13.6 ± 0.46	Test 1: 1.8 ± 0.32
	Test 2: 7.4 ± 0.53	Test 2: 12.4 ± 0.50	Test 2: 13.6 ± 0.36	Test 2: 13.6 ± 0.47	Test 2: 0.89 ± 0.43
	Test 3: 7.6 ± 0.57	Test 3: 11.7 ± 0.40	Test 3: 13.7 ± 0.35	Test 3: 13.7 ± 0.48	Test 3: 1.3 ± 0.38
Fat solution	Test 1: 7.5 ± 0.44	Test 1: 12.6 ± 0.39	Test 1: 13.5 ± 0.34	Test 1: 13.5 ± 0.43	Test 1: 1.1 ± 0.44
	Test 2: 7.2 ± 0.46	Test 2: 11.5 ± 0.43	Test 2: 13.7 ± 0.34	Test 2: 13.2 ± 0.46	Test 2: 0.54 ± 0.51
	Test 3: 6.9 ± 0.43	Test 3: 11.5 ± 0.50	Test 3: 13.5 ± 0.40	Test 3: 13.4 ± 0.52	Test 3: 0.77 ± 0.52
Placebo solution	Test 1: 8.2 ± 0.50	Test 1: 11.7 ± 0.42	Test 1: 13.1 ± 0.37	Test 1: 13.5 ± 0.47	Test 1: 1.5 ± 0.38
	Test 2: 7.1 ± 0.52	Test 2: 11.5 ± 0.41	Test 2: 13.2 ± 0.43	Test 2: 13.6 ± 0.53	Test 2: 1.2 ± 0.43
	Test 3: 7.8 ± 0.52	Test 3: 12.3 ± 0.43	Test 3: 13.4 ± 0.37	Test 3: 13.7 ± 0.49	Test 3: 1.3 ± 0.42

#### Discrimination

Placebo: The factor “time” had no significant influence on discrimination scores [*F*_(1, 2)_ = 1.6, *p* = 0.22].

Macronutrient and placebo solution: The factors “time”, “solution” and “time × solution” had no significant influence on discrimination scores [“time”: *F*_(1, 2)_ = 1.8, *p* = 0.19; “solution”: *F*_(1, 3)_ = 0.35, *p* = 0.76; “time × solution”: *F*_(2, 3)_ = 1.8, *p* = 0.16].

#### Identification

Placebo: The factor “time” had no significant influence on identification scores [*F*_(1, 2)_ = 1.0, *p* = 0.36].

Macronutrient and placebo solution: The factors “time”, “solution” and “time × solution” had no significant effect on subjects' identification scores [“time”: *F*_(1, 2)_ = 1.8, *p* = 0.19; “solution”: *F*_(1, 3)_ = 1.3, *p* = 0.29; “time × solution”: *F*_(2, 3)_ = 0.87, *p* = 0.47] (Table 6).

#### Intensity ratings

Placebo: The factor “time” had no significant influence on intensity ratings [*F*_(1, 2)_ = 0.98, *p* = 0.38].

Macronutrient and placebo solution: The factors “time”, “solution” and “time × solution' had no significant effect on subjects' intensity ratings [“time”: *F*_(1, 2)_ = 0.91, *p* = 0.39; “solution”: *F*_(1, 3)_ = 0.88, *p* = 0.44; “time × solution”: *F*_(2, 3)_ = 0.44, *p* = 0.76]; (Table 6).

#### Hedonic ratings

Placebo: The factor “time” had no significant influence on hedonic ratings [*F*_(1, 2)_ = 1.8, *p* = 0.19].

Macronutrient and placebo solution: The factors “time” and “solution” significantly affected hedonic ratings [“time”: *F*_(1, 2)_ = 9.9, *p* ≤ 0.001; “solution”: *F*_(1, 3)_ = 3.1, *p* ≤ 0.05] (Figure 5B), but “time × solution” had no significant effect on subjects' hedonic ratings [*F*_(3, 2)_ = 0.60, *p* = 0.66] (Table 6).

Isovolumetric conditions: *Post-hoc* analyses could not show any significant differences between the four solutions.

## Discussion

This is the first study to investigate the effect of different macronutrients on psychophysical, metabolic and olfactory function in parallel. Our study clearly confirmed our hypothesis that ingestion of different isocaloric macronutrients dissimilarly affects psychophysical and metabolic function due to different metabolism pathways. However, olfactory function was only affected by time. We could also show by correlation analyses that the parameter active ghrelin represents a predictive value for hunger and food craving.

Isocaloric macronutrients/Isovolumetric-isocaloric conditions: Comparing isovolumetric-isocaloric conditions (applied volume of 1500 mL/30 min, 600 kcal), we found that mood ratings were significantly lower directly after fat ingestion compared to the other macronutrient solutions (protein and carbohydrate). This can be easily explained by the high energy density as mentioned above and the common experience that digestion of a huge amount of fat is an exhausting process (Wells et al., 1995). Fischer et al. (2001) observed that mood was not influenced by food intake under normal conditions. A study by Smith et al. (1999) demonstrated that food consumption was associated with greater positive mood. However, the food applied in that study was low caloric and subjects had free choice of food. The type of solution showed a significant influence on the parameter hunger, i.e., participants rated their hunger feeling significantly lower after ingestion of protein compared to fat. Many studies demonstrated that fat ingestion has a relatively weak impact on satiety; as a consequence, a high fat diet leads to weight gain because more food has to be consumed to feel satiated (de Castro, 1987; Lissner et al., 1987; Bellisle et al., 1998). Our study did not prove these findings. However, Cecil et al. (1999) observed that ingestion of fat-rich food led to higher satiety compared to isocaloric and isovolumetric carbohydrate-rich food. An explanation could be the ingestion of isovolumetric nutrient solutions. Normally, fatty food has a higher energy density and thus a lower volume compared to carbohydrate and protein-rich food. Consequently, more fatty food has to be ingested to reach similar gastric distension compared to carbohydrate and protein-rich food. Besides, protein intake suppresses subsequent food consumption and has a higher satiating effect than fat and carbohydrate (de Castro, 1987; Poppitt et al., 1998). This is in line with our findings. In our experiments, the type of solution showed a significant influence on food craving, i.e., following carbohydrate intake vegetable craving was significantly higher compared to protein intake. Moreover, at the end of the test session, food craving was either comparable to the ratings directly before ingestion of the solution or even higher. At the end of test session, highest craving ratings occurred after carbohydrate intake, followed by fat intake, and protein intake showed lowest craving ratings compared to the ratings directly before ingestion of the solution. These results demonstrate that ingestion of different macronutrients leads to different food craving patterns, both regarding intensity and food type. It can also be argued that food craving patterns represent the motivation to consume different food. Hill et al. (Hill and Heaton-Brown, 1994) reported that food craving can be characterized as a hunger-modifying, mood-improving experience that is directed at wanting to consume highly pleasant-tasting food. In another study, Hill et al. (Hill and Weaver, 1991) demonstrated that food craving is very often associated with hunger. Our results support these findings because hunger and craving sensations showed similar patterns over time. Furthermore, at the end of test session, the different patterns of blood plasma levels of the solutions show that the hunger hormone levels were highest after carbohydrate intake, followed by fat intake, and protein intake showed lowest hunger hormone levels compared to the hunger hormone levels directly before ingestion of the nutrient solution. These findings are also in line with the observed hunger and food craving patterns of our study. Here, the most satiating macronutrient is protein, followed by fat while carbohydrate has the lowest effect on satiety.

The “hunger hormone” grehlin also influences regulating reward perception in dopamine neurons linking the ventral tegmental area to the nucleus accumbens (Naleid et al., 2005). Our study design allows to relate active ghrelin concentrations with hunger and craving ratings for each nutrient solution separately. We found a significant positive correlation of active ghrelin with hunger and fat, protein and sweets craving for each nutrient solution indicating that ghrelin could be a predictor of these parameters independent of nutrient solution used. However, active ghrelin significantly correlated with carbohydrate craving for carbohydrate and fat solution and with vegetable craving for fat solution only. These results show that the predictive value of active ghrelin could depend on the type of craving measured and macronutrient solution used.

Placebo/Isovolumetric conditions: Intake of a placebo solution (volume of 1,500 mL/30 min, 0 kcal) significantly influenced hunger and food craving. Directly after consumption of the placebo solution, hunger and food craving (fat, carbohydrate, sweets) ratings significantly decreased and increased thereafter. This phenomenon has to be solely associated with the distension of the stomach because the hunger-related metabolic parameters are not affected by ingestion of the placebo solution. This fact is clarified by the blood plasma levels of the hunger-inducing hormone active ghrelin and the precursor desacyl ghrelin: neither parameter shows a reduction after placebo intake. Regarding the effects of the placebo solution, a pure mechanical gastric distension study performed by Wang et al. (2008) supports our findings. The researchers demonstrated that mechanical distension of the stomach generates the perception of fullness. The effects of the placebo solution could also be the explanation of the results of a water preloading study performed by Parretti et al. (2015). The researchers demonstrated that water ingestion a short time before meal intake reduces hunger and leads to weight reduction. In our study, we also see a reduction in hunger and food craving ratings after ingestion of the placebo solution.

Isovolumetric conditions include the placebo condition (applied volume of 1,500 mL/30 min, protein, carbohydrate, fat and placebo solution) and we also investigated differences between 600 kcal of the macronutrient solutions and 0 kcal of the placebo solution. We found that mood ratings directly after placebo intake were significantly higher compared to fat intake. Macht et al. (2003) demonstrated that, with increasing energy density of food, negative emotions like anger, fear, sadness and shame are induced directly after consumption. This negative effect on mood is in line with our findings. Moreover, the type of solution showed a significant influence on the parameter hunger, i.e., participants rated their hunger feeling significantly higher after placebo intake compared to protein and carbohydrate intake. In our experiments, the type of solution showed a significant influence on the psychophysical parameter food craving, i.e., following placebo intake, fat, protein, carbohydrate and vegetable food craving was significantly higher than after protein intake. Furthermore, carbohydrate craving was significantly higher after placebo intake compared to carbohydrate intake. In addition, the different patterns of blood plasma levels of the solutions show that the hunger hormone levels were significantly higher regarding ingestion of placebo compared to protein. This effect seems to be related to the distention of the stomach as well as to the different nutrient intake. Wijlens et al. (2016) support our findings. The authors showed that a gastric infusion of 700 kcal increased satiety and lowered subsequent food intake by 35% compared to an isovolumetric gastric infusion of 100 kcal.

Cognition and Olfaction: Concerning cognition, our study showed that overall alertness ratings were significantly lower regarding protein intake compared to carbohydrate and placebo intake. In contrast, a study by Albrecht et al. (2009) demonstrated that there were no significant differences of alertness in hunger and satiated state. Furthermore, the error rate of alertness with alarm was significantly influenced by the factor “time × solution”, i.e., at time 3 (240–300 min after ingestion of protein) the error rate increased while it decreased regarding fat consumption compared to pre-intake.

Additionally, the incompatibility error rate was significantly influenced by the factor “time × solution,” i.e., at time 2 (60–120 min after ingestion of protein) the error rate was significantly lower compared to fat ingestion and at time 3 (240–300 min after ingestion of protein) the error rate was significantly lower compared to carbohydrate and placebo ingestion. Different researchers also reported discrepancies between carbohydrate-rich versus protein-rich food intake regarding cognition. They demonstrated that a carbohydrate-rich diet leads to worse results with respect to attention compared to a protein-rich diet 75–210 min post-ingestion (Spring et al., 1982; Lieberman et al., 1986; Smith et al., 1988). These findings are in line with our incompatibility error rate findings. In contrast to our results, comparing the cognitive performance 90–150 min after low/medium fat and high fat ingestion showed that subjects who consumed a high fat meal need more time to finish attention and declarative memory tasks (Lloyd et al., 1994; Smith et al., 1994; Wells et al., 1995). Within the analyzed time frames, it seems that different food intake affects just some cognitive functions. We found some negative effects of the protein solution regarding overall alertness and error rate of alertness with alarm. However, the overall alertness of protein did not significantly differ from the effects of fat in our study. Nevertheless, a larger negative impact on cognitive functioning was described for high fat diets (Lloyd et al., 1994; Smith et al., 1994; Wells et al., 1995). In order to explain this discrepancy more specific studies employing comparable cognitive testing batteries are required. These studies should focus on the effects of single ingestions of nutrients as well on the effects of long term diets.

Comparing the four different solutions, we found in terms of olfaction a significant effect of the factor “time” for *n*-butanol threshold detection. Our study demonstrated that independent of ingested solution, threshold scores were lower at time 3 (240–300 min after ingestion of the solutions) compared to pre-intake. Concerning the macronutrient solutions our findings are in line with Guild et al. (Guild, 1956), who observed that olfactory sensitivity regarding coffee odor was highest before and least after satisfying meals. Hedonic evaluation showed a significant effect of “time,” i.e., odorants were perceived as more unpleasant directly after ingestion of the solutions; this was independent of solution type. Regarding the macronutrient solutions, our findings are in line with Albrecht et al. (2009), who reported decreased pleasantness of a food-related odorant during satiety compared to hunger state. Further, Rolls et al. (Rolls and Rolls, 1997) observed that eating food to satiety tended to decrease pleasantness ratings after food consumption.

## Conclusion

Intake of a placebo solution significantly reduced hunger and food craving directly after consumption. This phenomenon seems to be solely associated with the distension of the stomach because the hunger-related metabolic parameters are not affected by ingestion of the placebo solution. The isovolumetric condition showed that the effects on hunger and food craving are not only related to the distention of the stomach but also emphasizes the importance of the type of macronutrient applied. Moreover, hunger and food craving ratings and hunger hormone levels demonstrated that the hierarchical order that appears in satiating efficiencies of isovolumetric-isocaloric ingested macronutrients is protein > fat > carbohydrate. Consequently, our results confirm that protein ingestion achieves the best satiating effect of all macronutrients.

The significant correlations of active ghrelin concentrations with hunger and craving ratings recommend ghrelin as predictor of these physiological measures. Under our experimental conditions active ghrelin concentrations predict hunger and fat, protein and sweets craving in a nutrient solution unspecific manner. In contrast, the predictive value of active ghrelin for carbohydrate and fat craving was specific to the nutrient solutions carbohydrate and fat.

Limitations of our pilot study are the relatively small number of subjects and the fact that only male participants were included. To confirm our preliminary results, further studies with higher numbers of male and female participants are required. Beyond the scope of food craving, hunger and satiety, future studies should also investigate the effects of different oral intake rates at a behavioral level, e.g., on subsequent food consumption.

The hierarchical order of satiating efficiency for the different macronutrients tested and the predictive value of active ghrelin concentrations for hunger and food craving could help to improve the management of hunger and food craving during artificial feeding and during oral diets.

## Author contributions

Each author has participated sufficiently in the work, intellectually or practically, to take public responsibility for the content of this article, including the conception, design, and conduct of the experiment and data analysis and interpretation. SB, MD and JW were responsible for subject recruiting. SB and MD carried out the practical work and were responsible for data analysis. AB, AD, CS, GM, JK, and NT conceived the study, and SB, MD, JW, and MF participated in the design of the study. All authors contributed to the manuscript and approved the final version.

### Conflict of interest statement

The authors declare that the research was conducted in the absence of any commercial or financial relationships that could be construed as a potential conflict of interest.

## Abbreviations

NPY: neuropeptide Y
AgRP: agouti-related protein
α-MSH: α-melanocyte stimulating hormone
CART: cocaine- and amphetamine regulated transcript
CCK: cholecystokinin
PYY: peptide YY
SCID: structured clinical interview for DSM-IV
BDI: Beck-Depressions-Inventar
FEV: Fragebogen zum Essverhalten
VAS: visual analog scale.
